# Primary dengue virus infections induce differential cytokine production in Mexican patients

**DOI:** 10.1590/0074-02760150359

**Published:** 2016-03

**Authors:** Sergio Isaac de la Cruz Hernández, Henry Nelson Puerta-Guardo, Hilario Flores Aguilar, Silvia González Mateos, Irma López Martinez, Vianney Ortiz-Navarrete, Juan E Ludert, Rosa María del Angel

**Affiliations:** 1 Instituto de Diagnóstico y Referencia Epidemiológicos, Departamento de Virología, México DF, México; 2 Centro de Investigación y Estudios Avanzados, Instituto Politécnico Nacional, Departamento de Infectómica y Patogénesis Molecular, México DF, México; 3 Instituto de Diagnóstico y Referencia Epidemiológicos, Departamento de Inmunología e Inmunogenética, México DF, México; 4 Centro de Investigación y de Estudios Avanzados, Instituto Politécnico Nacional, Departamento de Biomedicina, México DF, México

**Keywords:** dengue haemorrhagic fever, dengue pathogenesis, cytokines

## Abstract

Severe dengue pathogenesis is not fully understood, but high levels of proinflammatory cytokines have been associated with dengue disease severity. In this study, the cytokine levels in 171 sera from Mexican patients with primary dengue fever (DF) and dengue haemorrhagic fever (DHF) from dengue virus (DENV) 1 (n = 116) or 2 (n = 55) were compared. DF and DHF were defined according to the patient’s clinical condition, the primary infections as indicated by IgG enzymatic immunoassay negative results, and the infecting serotype as assessed by real-time reverse transcription-polymerase chain reaction. Samples were analysed for circulating levels of interleukin (IL)-12p70, interferon (IFN)-γ, tumour necrosis factor (TNF)-α, IL-6, and IL-8 using a commercial cytometric bead array. Significantly higher IFN-γ levels were found in patients with DHF than those with DF. However, significantly higher IL-12p70, TNF-α, and IL-6 levels were associated with DHF only in patients who were infected with DENV2 but not with DENV1. Moreover, patients with DF who were infected with DENV1 showed higher levels of IL-12p70, TNF-α, and IL-6 than patients with DHF early after-fever onset. The IL-8 levels were similar in all cases regardless of the clinical condition or infection serotype. These results suggest that the association between high proinflammatory cytokine levels and dengue disease severity does not always stand, and it once again highlights the complex nature of DHF pathogenesis.

Dengue virus (DENV) infection is the most important arthropod-borne viral disease of public health significance, with an estimate of over 50 million infections occurring each year and resulting in more than 24 thousand deaths (Guha-Sapir & Schimmer 2005). DENV is a member of the Flaviviridae family, genus Flavivirus, and it is transmitted to humans through the bite of*Aedes* mosquitoes, primarily *Aedes aegypti* (Clyde et al. 2006, Noisakran & Perng 2008, Martina et al. 2009). An infection with any one of the four DENV serotypes may be asymptomatic or may result in different clinical symptoms, ranging from a febrile illness known as dengue fever (DF) to the most severe form of the illness, which is known as dengue haemorrhagic fever (DHF). DF is associated with fevers, headaches, myalgia, bone/joint pain, and rashes. DHF presents all the symptoms of DF in combination with two major pathophysiological hallmarks that determine the severity of disease, namely bleeding and plasma leakage, as a result of increased vascular permeability and abnormal haemostasis. The loss of vascular fluids may result in dengue shock syndrome (DSS), which is a form of hypovolaemic shock that is characterised by haemoconcentration. If appropriate care is not given, this shock might result in the death of the patient (Lei et al. 2008, Noisakran & Perng 2008, Martina et al. 2009).

Despite the existence of many relevant studies, the pathogenesis of DHF/DSS is not fully understood yet. The virulence hypothesis points to differences in DENV strains as being responsible for differences in disease severity (Leitmeyer et al. 1999). The immune hypothesis notes that individuals with secondary infections (caused by a heterologous DENV serotype) have a higher risk of developing DHF/DSS. The presence of cross-reactive, nonneutralising antibodies from the primary infection result in antibody-dependent enhancement (ADE), which is believed to augment viral replication by increasing the number of Fc receptor bearing cells that are infected during secondary DENV infection (Halstead 1970, Halstead & O’Rourke 1977,Brandt et al. 1982). This condition results in the activation of pre-existing cross-reactive T-lymphocytes and the release of inflammatory cytokines and cellular mediators, thus leading to the increased plasma leakage characteristic of DHF/DSS (Kurane & Ennis 1997). In some epidemic situations, DHF cases among patients with primary infections have been documented (Barnes & Rosen 1974, Scott et al. 1976, Rosen 1977), and even in primary cases, the progress from DF to DHF has been associated with increased levels of tumour necrosis factor (TNF)-α, interleukin (IL-1), and IL-6 (Iyngkaran et al. 1995). Thus, abundant evidence suggests that high cytokine levels have a role in DHF development (Braga et al. 2001,Nguyen et al. 2004, Perez et al. 2004, Bozza et al. 2008, Restrepo et al. 2008a, b).

In this study, we measured the cytokine levels in sera samples from Mexican patients who were affected by primary infections but showed a high number of DHF cases in comparison with DF cases. The analysis of primary cases only offers the opportunity to compare both clinical conditions, avoiding other factors that may be associated with secondary infections. The results show higher levels of interferon (IFN)-γ in patients with DHF than patients with DF. However, only patients who were infected with DENV2 and not with DENV1 showed higher levels of IL-12p70, TNF-α, and IL-6 associated with DHF. The results suggest that the association between higher proinflammatory cytokine levels and DHF does not always stand, and they highlight the complex nature of the pathogenesis of this clinical condition.

## SUBJECTS, MATERIALS AND METHODS

*Clinical samples* - A total of 171 human serum samples were provided by the Institute of Epidemiological Diagnosis and Reference (InDRE), in the city of Mexico, Mexico. All epidemiological data were obtained with a clinical history to accompany each sample. Sera samples were either collected from states from the north-western region of the country, where serotype 1 was circulating at the time, or from the southern states, where serotype 2 was circulating. The cases were defined as DF or DHF by following clinical criteria based on the traditional World Health Organization classification from 1997, as detailed by Navarrete-Espinosa et al. (2005). Clinical practice in Mexican public centres for febrile patients suspected of having dengue consist of a blood sample collection before the administration of any treatment; thus, the samples used in this study came from untreated patients. The samples were assayed by commercial ELISA (PLATELIA™, BIO-RAD, France) for soluble DENV nonstructural protein-1 (NS1) and by quantitative reverse transcription-polymerase chain reaction (RT-PCR) to determine the infecting serotype (de la Cruz-Hernández et al. 2013). The cases were classified as primary or secondary according to a Dengue IgM and IgG Capture ELISA from PanBio (Australia). Because all the serum samples corresponded to the acute phase of the disease (they were collected between 0-5 days after the onset of fever), a sample was classified as a primary infection when it was negative for IgG and as a secondary infection when positive for IgG (de la Cruz-Hernández et al. 2013). Samples were transported on ice and kept frozen at -70ºC until they were assayed for cytokines.

The panel consisted of 116 DENV1-positive samples (DF n = 60, DHF n = 56) and 55 DENV2-positive samples (DF n = 41, DHF n = 14). These samples corresponded to the acute period of infection, and they were all taken from patients during the first five days post-fever onset. Study day zero was defined as the day when the patient began to have a fever. With respect to age and gender, 82 (48%) of the samples corresponded to female patients and 89 corresponded to male patients; 60% of the samples (n = 103) were collected from patients between 0-25 years of age.

*Cytokine assays* - The levels of IL-12p70, TNF-α, IL-6, IL-8, and IFN-γ were determined using a Cytometric Bead Array (BD Biosciences, USA) according the manufacturer´s instructions with the support of the FACS Calibur-E3318 Flow Cytometer System. The cytokine levels were quantified using FCAP Array software v.3.0 (BD Biosciences). Five negative sera from healthy blood donors that tested negative for dengue were included, in addition to the control reagents included in the array, for the standardization process.

*Statistical analysis* - The Mann-Whitney *U* test was used to compare the median values between groups. Graphs were generated with GraphPad Prism 5 software.

*Ethics* - Because it is obligatory to report dengue in Mexico, informed consent was not required; nevertheless, patient confidentiality was conserved and the samples were used solely for research purposes. This study was approved by the Research Committee of the InDRE.

## RESULTS

All 171 sera used in this study were found to be positive for DENV NS1 by ELISA and they all corresponded to primary infections (i.e., they were negative for IgG; there were less than 22 PanBio units for IgG). The real-time RT-PCR results indicated that 116 samples corresponded to infections caused by DENV1 (60 DF cases and 56 DHF cases) and 55 sera corresponded to infections by DENV2 (41 DF cases and 14 DHF cases).

Significantly increased levels of the cytokines IFN-γ, TNF-α, and IL-8, but not IL-6 and IL-12p70, were found in patients with DF or DHF in comparison with those of healthy blood donors (Table I). In addition, significantly higher IFN-γ levels were found in DHF cases than in DF cases for patients infected with DENV1 [DHF median = 45.1 pg/mL; 25-75% interquartile range (IQR) = 42.8-53.1 pg/mL; range = 40.3-120 pg/mL in comparison with the DF median = 44.2 pg/mL; 25-75% IQR = 41.9-46.4 pg/mL; range = 40.3-95.6 pg/mL; p = 0.0128] and DENV2 (DHF median = 50.1 pg/mL; 25-75% IQR = 44.2-60.3 pg/mL; range = 42.5-73.6 pg/mL in comparison with the DF median = 45.5 pg/mL; 25-75% IQR = 42.1-49.3 pg/mL; range = 39.2-64.1 pg/mL; p = 0.0351). However, the IL-12p70, TNF-α, and IL-6 levels were higher in DHF than DF cases, but only in patients infected with DENV2 but not with DENV1 (Figure). There were no differences in the IL-8 levels among DF and DHF patients, whether they were infected with DENV1 or DENV2. Notably, patients with DHF who were infected with DENV2 showed significantly higher levels of IL-12p70, TNF-α, and IL-6 than patients with DHF who were infected with DENV1. Higher IFN-γ and IL-8 levels were also observed, but the trend was not significant (Supplementary Table).

**Figure:**
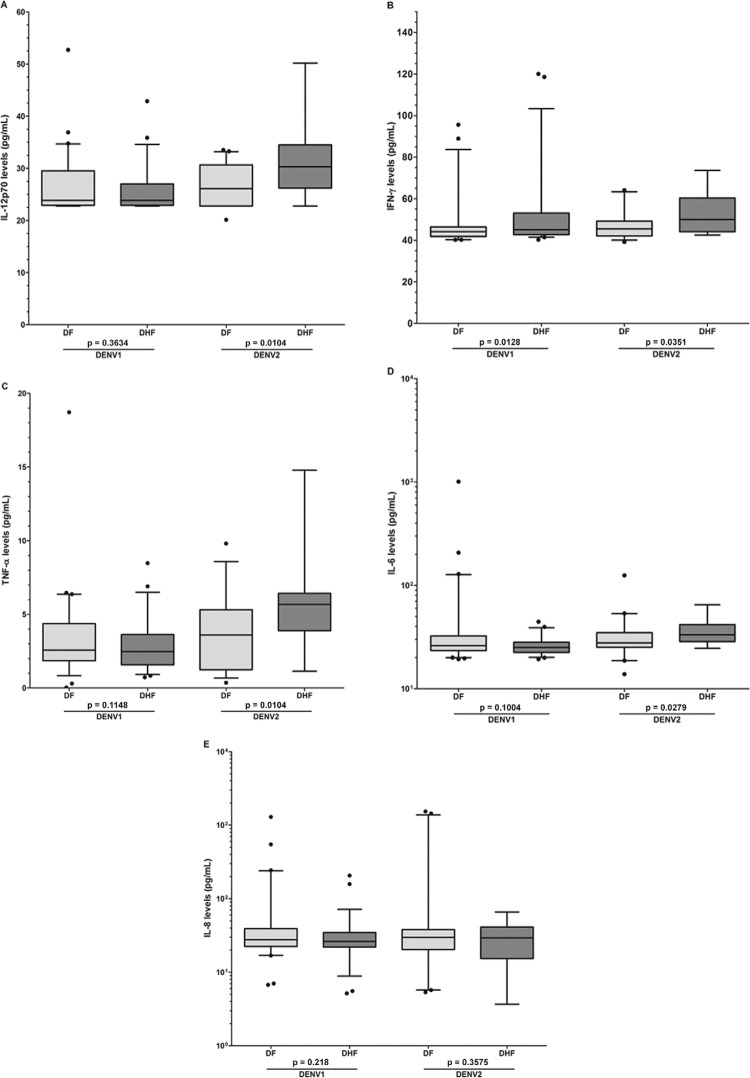
Levels of cytokines in clinical samples of dengue virus (DENV) serotype 1 or 2 infected patients. Concentrations (pg/mL) of cytokines were determined by a commercial cytometric bead array. Each cytokine is presented in a different panel for clarity. Box-and-whisker plots show median values (horizontal line in the box), 25-75% interquartile range (upper-lower limits of the box), 95% range of data (error bars), and outliers (black circles). Statistical significance was p ≤ 0.05. DF: dengue fever; DHF: dengue haemorrhagic fever; IL: interleukin.

**TABLE I:** Cytokines median levels (pg/mL) in healthy donors and patients with dengue disease

Cytokine	Healthy (n = 5)	DF (n = 101)	DHF (n = 70)	p^*a*^	p^*b*^
IL-12p70	25.2 (23.0-25.5)	24.6 (20.1-52.7)	25.0 (22.7-50.1)	NS	NS
IFN-g	39.2 (39.2-42.4)	44.6 (39.2-95.6)	45.7 (40.2-120)	0.0006	0.0002
TNF-a	1.1 (0.001-1.4)	2.7 (0.03-18.7)	2.7 (0.72-14.7)	0.0099	0.0089
IL-6	22.7 (16.9-27.9)	26.9 (13.8-1009)	25.8 (19.3-64.9)	NS	NS
IL-8	7.2 (6.0-13.7)	29.4 (5.3-1535)	26.6 (3.6-206)	0.0003	0.0006

*a*: healthy vs. dengue fever (DF);*b*: healthy vs. dengue haemorrhagic fever (DHF). Median (minimum-maximum), significance p ≤ 0.05. IFN: interferon; IL: interleukin; NS: nonsignificant; TNF: tumour necrosis factor.

Because the development of dengue disease has been shown to be a dynamic process (Iyngkaran et al. 1995, Tricou et al. 2011, Popper et al. 2012), cytokine levels were determined in samples that were collected zero-two and three-five days after fever onset (Table II). Samples collected five days after fever onset were not available because they are not collected as part of the Mexican dengue survey program. In sera collected zero-two days after fever onset from patients infected with DENV1, significantly lower levels of IL-12p70, TNF-α, and IL-6 were found in DHF than in DF patients. Interestingly, the significant differences observed for TNF-α levels between DF and DHF cases that were observed in early collection sera samples (0-2 days after fever onset) were no longer observed in sera samples that were collected later (3-5 after fever onset); the opposite was observed for IFN-γ, reflecting the dynamic nature of cytokine levels in patients over the course of the disease.

**TABLE II:** Cytokines median levels (pg/mL) in patients with dengue according to the days of disease

		0-2 days		p	3-5 days		p
	
		DF	DHF		DF	DHF	
IL-12p70	DENV1	26.6 (22.7-52.7)	23.8 (22.7-35.8)	0.056	23.1 (22.7-36.8)	23.6 (22.7-42.8)	NS
	DENV2	26.1 (20.1-33.5)	29.3 (23.9-32.6)	NS	26.4 (22.7-32.4)	31.8 (22.7-50.1)	0.034
IFN-g	DENV1	44.2 (40.3-50.7)	44.7 (40.2-120.0)	NS	43.4 (40.2-95.6)	45.4 (41.5-118)	0.042
	DENV2	45.6 (39.2-64.1)	46.1 (42.5-73.6)	NS	44.7 (40.2-58.5)	51.9 (44.5-68.2)	0.026
TNF-a	DENV1	3.9 (0.03-18.7)	2.4 (0.94-6.3)	0.009	2.0 (0.83-6.3)	2.4 (0.72-8.4)	NS
	DENV2	3.6 (0.36-9.8)	5.3 (3.7-6.8)	0.055	3.1 (0.72-8.3)	6.2 (1.1-14.7)	NS
IL-6	DENV1	28.0 (21.2-128)	25.1 (19.3-44.4)	0.01	24.4 (19.3-1009)	25.0 (19.9-39.6)	NS
	DENV2	29.2 (13.8-124)	30.0 (24.6-64.9)	NS	26.8 (18.6-46.4)	35.6 (30.8-50.4)	0.006
IL-8	DENV1	28.7 (6.7-547)	27.2 (5.1-48.4)	NS	26.8 (16.9-1293)	25.5 (9.4-206)	NS
	DENV2	29.7 (5.3-1535)	36.6 (3.6-66.0)	NS	29.9 (16.3-226)	27.8 (9.0-40.9)	NS

median (minimum-maximum), significance p ≤ 0.05. Dengue virus (DENV) 1 [0-2 days: dengue fever (DF) = 26, dengue haemorrhagic fever (DHF) = 25; 3-5 days: DF = 34, DHF = 31]; DENV2 (0-2 days: DF = 25, DHF = 7; 3-5 days: DF = 16, DHF = 7). IFN: interferon; IL: interleukin; NS: nonsignificant; TNF: tumour necrosis factor.

An analysis of cytokine levels according to gender (males, n = 89, and females, n = 82) or age (0-25, n = 103, and over 25 years old, n = 68), and clinical condition (DF vs. DHF) did not show clear evidence explaining the role of either of these two parameters in serum cytokine levels (data not shown).

## DISCUSSION

The major pathophysiological hallmarks that distinguish DHF from DF are bleeding and plasma leakage, which result from increased vascular permeability and abnormal haemostasis (Lei et al. 2008, Noisakran & Perng 2008, Martina et al. 2009). Because of the lack of structural damage, the short-lived nature of the plasma leakage syndrome and the remarkably rapid recovery of children with DSS, soluble mediators, namely cytokines, have been determined to be responsible for permeability alterations (Innis 1995). The relations between the development of severe disease from DF to DHF in primary DENV infection and the sequential changes in the TNF-α, IL-1, and IL-6 levels (overall on day 6, when the signs and symptoms of hypovolaemic shock appeared) along with the full recovery suggests the participation of these cytokines in the pathogenesis of the disease (Iyngkaran et al. 1995). The presence of significantly higher levels of pro-inflammatory cytokines in sera from either DF or DHF patients in comparison with healthy subjects is well established (Braga et al. 2001, Nguyen et al. 2004, Chakravarti & Kumaria 2006, Priyadarshini et al. 2010, Mangione et al. 2014). However, because of difficulties in making direct study comparisons and the existence of contradictory results, the role of pro and antiinflammatory cytokines during DHF pathogenesis in comparison with that of DF is not well understood. Differential TNF-α, IFN-γ, IL-6, and IL-8 levels have been found when comparing DHF and DF patients. Several studies have shown higher levels of IFN-γ in DF patients than in DHF patients (Chakravarti & Kumaria 2006, Priyadarshini et al. 2010,Mangione et al. 2014, Soundravally et al. 2014), and others have reported higher levels in DHF patients or no differences in this cytokine, along with the levels of IL-12, TNF-α, IL-6, and IL-8 in comparison with those of DF patients (Juffrie et al. 2000, Braga et al. 2001, Chakravarti & Kumaria 2006, Chen et al. 2006, Bozza et al. 2008, Priyadarshini et al. 2010,Rathakrishnan et al. 2012, Mangione et al. 2014, Soundravally et al. 2014, Ferreira et al. 2015, Pandey et al. 2015).

Although it is limited in its number of cases, this study offers the advantage of allowing for the direct comparison of cytokine levels among patients affected by DF or DHF only during their primary infection, when the disease was caused by two different DENV serotypes. Our results show higher levels of IFN-γ in patients with DHF than DF. However, the proinflammatory cytokines IL-12p70, TNF-α, and IL-6 were found to be elevated in DHF in relation to those in DF, but only in patients infected with DENV2. For patients infected with DENV1, the TNF-α, IL-12p70, and IL-6 levels were higher in DF than in DHF patients. This finding is remarkable, especially for TNF-α, which is a classical proinflammatory cytokine that is implicated in increased vascular permeability and is frequently found to be augmented in patients with DHF/DSS (Braga et al. 2001, Nguyen et al. 2004, Angelini et al. 2006, Soundravally et al. 2014, Vervaeke et al. 2015). However, contradictory data for the role of TNF-α in severe dengue has also been observed in genetic studies, in which a TNF-α gene polymorphism (-308A allele) has been associated not only with severe disease, but also with protective effects against DHF (Fernández-Mestre et al. 2004, Perez et al. 2010, Chuansumrit et al. 2013,Sam et al. 2015). Moreover, the observation that suggests that the infecting serotype may have a role in the induction of cytokines deserves further analysis. The higher cytokine levels observed for DENV2-infected patients did not correlate with higher viraemia levels in those patients, and yet a tendency towards higher NS1 circulating levels was observed (data not shown).

The “original antigenic sin” hypothesis suggests that individuals with secondary infections have increased risks of developing DHF/DSS because of the activation of pre-existing cross-reactive T-lymphocytes and because of the presence of cross-reactive nonneutralising antibodies. These conditions favour the infection of highly susceptible FcR-bearing cells, and they all ultimately lead to increased releases of pro-inflammatory cytokines and cellular mediators related to plasma leakage, which is the hallmark of severe dengue (Halstead 1970, Halstead & O’Rourke 1977, Brandt et al. 1982, Kurane & Ennis 1997, Mongkolsapaya et al. 2003, Rothman 2011). However, the “original antigenic sin” hypothesis fails to explain DHF cases during primary infections (Barnes & Rosen 1974, Scott et al. 1976, Rosen 1977). In addition, the use of immunosuppressive drugs in clinical trials was aimed at controlling abnormal homeostasis in children with severe dengue, and it resulted in no improvement in clinical conditions, suggesting that mechanisms other than those mediated by T-cells are playing a role in plasma leakage (Halstead 2013). Moreover, the direct participation of soluble NS1 in plasma leakage was recently documented (Beatty et al. 2015, Modhiran et al. 2015). Cytokine levels in primary and secondary DENV infections have been compared, and the differences are not enough to indicate that DHF pathogenesis is completely attributable to increased cytokine expression during secondary DENV infection, despite their importance in endothelial damage and inflammatory responses (Chakravarti & Kumaria 2006, Priyadarshini et al. 2010). There is also evidence to suggest that the cytokine expression levels decreased in subsequent DENV infections (Sierra et al. 2012). In this study, only primary DENV infections were considered, and the higher levels of most of the analysed cytokines were observed in samples from DHF patients who were infected with DENV2. These samples were collected from three-five days after fever onset. Nonetheless, for the samples collected from zero-two days after fever onset, significantly higher levels of IL-12p70, TNF-α, and IL-6 cytokines were observed in DF relative to DHF patients infected with DENV1.

The histories of exposure, as well as age and gender have been reported to influence cytokine expression levels. School-age children may have a more robust immune response to DENV that in some cases contributes to the disease severity (Vaughn et al. 2000). However, our data did not show differences in sera cytokine levels among patients according to their gender or age. In this study, women with DHF caused by DENV2 showed significantly higher levels of IL-12p70 and TNF-α, but not IFN-γ and IL-6, and the opposite trend was observed for men. Likewise, patients in the 0-25 age range with DHF showed significantly higher levels of IFN-γ, IL-12p70, and TNF-α, and patients over 25 years of age displayed significant differences only for IL-6.

In summary, these results suggest that although proinflammatory cytokines such as IL-12p70, IFN-γ, TNF-α, and IL-6 may actually be part of the processes that lead to plasma leakage, other factors such as viraemia, antibody levels related to ADE or to molecular mimicry, infection history (primary or secondary infection), genetic background, and even age and sex also play a role in and highlight the complex nature of severe dengue pathogenesis.

## Supplementary data

**TABLE:** Cytokines median levels (pg/mL) in patients with dengue haemorrhagic fever (DHF) according to dengue virus (DENV) serotype

Cytokine	DHF^*a*^ (n = 56)	DHF^*b*^ (n = 14)	p
IL-12p70	23.8 (22.7-42.8)	30.3 (22.7-50.1)	0.0003
IFN-g	45.1 (40.2-120)	50.0 (42.5-73.6)	NS
TNF-a	2.4 (0.72-8.4)	5.6 (1.1-14.7)	0.0007
IL-6	25.1 (19.3-44.4)	33.1 (24.6-64.9)	< 0.0001
IL-8	26.2 (5.1-206)	29.3 (3.6-66.0)	NS

*a*: DHF patients infected y DENV1;*B*: DHF patients infected y DENV2. Median (minimum-maximum), significance p ≤ 0.05. IFN: interferon; IL: interleukin; NS: nonsignificant; TNF: tumour necrosis factor.
